# Clinical Quality Considerations when Using Next-Generation Sequencing (NGS) in Clinical Drug Development

**DOI:** 10.1007/s43441-021-00308-6

**Published:** 2021-05-27

**Authors:** Timothé Ménard, Alaina Barros, Christopher Ganter

**Affiliations:** 1 F. Hoffmann-La Roche AG, Basel, Switzerland; 2 Genentech Inc. - A Member of the Roche Group, South San Francisco, USA

**Keywords:** Clinical quality, Quality assurance, Next-generation sequencing, Genomics, Genetics, Clinical trials

## Abstract

Next-generation sequencing (NGS) and decreased costs of genomic testing are changing the paradigm in precision medicine and continue to fuel innovation. Integration of NGS into clinical drug development has the potential to accelerate clinical trial conduct and ultimately will shape the landscape of clinical care by making it easier to identify patients who would benefit from particular therapy(ies) and to monitor treatment outcomes with less invasive tests. This has led to an increased use of NGS service providers by pharmaceutical sponsors: to screen patients for clinical trials eligibility and for patient stratification, expanded Companion Diagnostic (CDx) development for treatment recommendations and Comprehensive Genomic profiling (CGP). These changes are reshaping the face of clinical quality considerations for precision medicine. Although some clinical quality considerations do exist in Health Authorities (HA) guidances and regulations (e.g., International Conference of Harmonization Good Clinical Practices—GCP), there is currently no holistic GxP-like detailed framework for pharmaceutical sponsors using NGS service providers in clinical trials, or for the development of CDx and CGP. In this research, we identified existing and applicable regulations, guidelines and recommendations that could be translated into clinical quality considerations related to technology, data quality, patients and oversight. We propose these considerations as a basis for pharmaceutical sponsors using NGS service providers in clinical drug development to develop a set of guidelines for NGS clinical quality.

**Supplementary Information:**

The online version contains supplementary material available at 10.1007/s43441-021-00308-6.

## Background

Next-generation sequencing (NGS) refers to large-scale, fast and efficient DNA (and RNA) sequencing technology. For DNA sequencing all NGS platforms perform sequencing of millions of small fragments of DNA in parallel (also called “massively parallel” or “deep” sequencing) and then rely on bioinformatic analysis against a reference genome to infer useful insights from detected variations. This technology can be utilized in many research areas, from molecular biology to human genetics, but is also used as part of clinical care in various therapeutic areas (e.g., in oncology) [1–4]. NGS and decreased costs of genomic testing are changing the paradigm in precision medicine and continue to fuel innovation, with new targets being identified, new therapies being developed and new indications being approved, for example in oncology [1–4]. Integration of NGS into clinical drug-development has the potential to accelerate clinical trial conduct and ultimately will shape the landscape of clinical care by making it easier to identify patients who would benefit from particular therapy(ies) and to monitor treatment outcomes with less invasive tests [5]. This has led to an increased use of NGS service providers (i.e., vendors that provide NGS services to pharmaceutical sponsors, also referred as NGS providers or NGS partners), to screen patients for clinical trials eligibility and for patient stratification [3, 4, 6] and develop Companion Diagnostics (CDx) for treatment recommendations and Comprehensive Genomic profiling (CGP) [7]. These changes are reshaping the face of clinical quality considerations for precision medicine.

In 2013, an opinion published in *Nature Medicine* [8] provided rationale for "*Establish[ing] good genomic practice to guide medicine forward*". The scope was broad, from analytical to clinical validation of NGS, but also included ethical considerations. Furthermore, it provided only high level principles that needed to be further developed and explained. Although some clinical quality considerations do exist in Health Authorities (HA) guidances and regulations (e.g., International Conference of Harmonization Good Clinical Practices—GCP [9]), there is currently no holistic GxP-like detailed framework for pharmaceutical sponsors using NGS service providers in clinical trials, or for the development of CDx and CGP.

In this review, we aimed to identify existing and applicable regulations, guidelines and recommendations that could be consolidated into clinical quality considerations for pharmaceutical sponsors using NGS service providers in clinical development. Integration of clinical quality standards for NGS can lead to unified standards for the generation of high quality data, ethical use of data with increased transparency of testing limitations and ultimately better outcomes for patients. Through this review we sought to highlight requirements (i.e., regulation, ISO standards, etc.) that are present, and to provide pragmatic GxP recommendations for further consideration when no requirement was available.

The scope of this review included:

- clinical quality aspects of data primarily generated by NGS service providers, and direct variables derived from NGS data
- use of these NGS data in the context of clinical trials (i.e., research involving human subjects)
- use of NGS for development of solutions to guide treatment recommendations, which are subject to regulatory approval, for example CDx and CGP

We considered the following to be out of scope:

- NGS methods and standards, except elements relevant for pharmaceutical sponsors clinical quality oversight
- Clinical quality aspects for secondary use of (genomic) data
- Applicable Good Manufacturing Practices (GMP) regulations
- Investigational Device regulations
- Local/national laws or regulations for clinical laboratories (i.e., Clinical Laboratory Improvement Amendments of 1988 (CLIA))

Although out of scope, this review will touch upon relevant ethical topics in using genomic data in clinical development, but will not provide detailed considerations. NGS use by pharmaceutical sponsors is not restricted to a specific therapeutic area, but increased use of precision medicine in oncology presents a significant benefit for patients. For example advances in the use of circulating tumor DNA (ctDNA) technologies offer less invasive testing options for HealthCare Providers (HCPs) to monitor disease progression or patients in high risk populations. Additionally development of new CDx's helps balance treatment risks by identifying targeted treatment options based on genomic profiles [5]. Genomics and precision medicine are fast evolving fields; hence relevant articles and guidance issued since 2010 were considered with the exception of foundational HA guidelines and regulations that still applied at the time of this publication.

## Methods

**Fig. 1:**
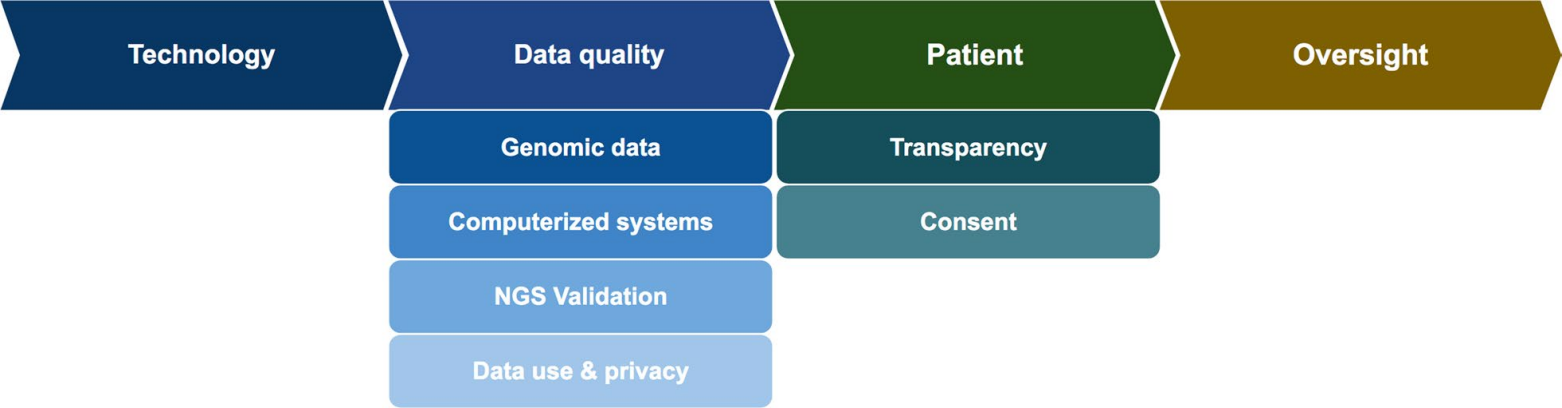
Key clinical quality considerations

We started by examining the NGS landscape using case studies to identify risk areas related to ensuring safety/safeguarding patients and data integrity in clinical trials. Through this examination we highlighted key clinical quality risk factors across the NGS lifecycle and grouped them into technology, data quality, patients (that undergo tumor testing) and oversight (of NGS service providers contracted by pharmaceutical sponsors) considerations (Fig. 1).

While this paper was not intended to be a systematic review, we applied the following strategy and criteria to screen and identify relevant regulations, standards and scientific journals.

- We focused on standards and regulations applicable to the International Conference on Harmonization (ICH) regions (i.e., United States of America (USA), European Union (EU) and Japan).
- From the ICH guidelines, we selected the one applicable to clinical drug development (i.e., which was the scope of this review)—ICH-E6 Good Clinical Practices [9]
- From ISO standards, we considered the ones relevant for Quality Management Systems (QMS) and the use of medical devices.
- We reviewed major HA regulations and guidance, and selected the ones that pertain to the development, use and validation of NGS in the context of use in clinical trials.
- We also included major HA regulations applicable to clinical drug development, for example the US Code of Federal Regulations (CFR) that encompass requirements for quality management, use of data and validation of electronic systems in clinical trials.
- Finally, we reviewed a selection of genetics scientific journals for relevant articles and reviews that could inform clinical quality considerations. We only considered scientific journals where institutions (such as the American College of Medical Genetics [10]) or scientific working groups were known to have discussed or issued recommendations regarding use of NGS in clinical practice.
- As explained in the Background section, we limited our search up to 2010 as NGS was an emerging technology and did not want to include outdated information.
- A list of standards, regulations and journals included for review can be found in the Supplementary Material.

To identify relevant information for clinical quality considerations, we applied inclusion/exclusions criteria to all documents screened and reviewed (see Table 1).

**Table 1:** Identification of relevant topics for clinical quality considerations for NGS in clinical development

Clinical quality risk factor	Inclusion	Clinical quality considerations
Data integrity	Yes	Technology, Data quality
Patient safety/well-being	Yes	Patients, Data quality
Applicability for pharmaceutical sponsors	Yes	Technology, Data Quality, Patients, Oversight
Applicability for clinical quality organization	Yes	Technology, Data Quality, Patients, Oversight
Relevance for patients, investigators and healthcare providers (HCPs)	Yes	Patients
NGS methods and standards	No	Data quality (if/when applicable)
NGS analytical validation	No	Not applicable
Good manufacturing practices	No	Not applicable
Secondary use of NGS data	No	Not applicable

We then translated the outcomes of the review into a set of clinical quality considerations, grouped by appropriate topic: technology, data quality, patient (that undergo tumor testing) and oversight (of NGS service providers contracted by pharmaceutical sponsors). These topics were based on the respective needs of various quality assurance professionals within a pharmaceutical sponsor organization (the primary audience for this review), for example these considerations can be leveraged to:

- establish expectations for sponsor oversight of service providers;
- to efficiently plan and conduct risk-based audit programs;
- to evidence data integrity and patient safety was maintained in clinical trials; and/or
- clearly communicate the state of NGS clinical quality to internal and external stakeholders.

As this was not intended to be a systematic review, we acknowledge that the scope of the search strategy could have been broader; therefore, this can be considered as the main limitation of this review.

## Clinical Quality Considerations: Technology

Bioinformatics algorithms executed in a predefined sequence to process NGS data are collectively referred to as an NGS bioinformatics pipeline [11]. This first set of clinical quality considerations applies to the use of technology in such NGS bioinformatics pipelines. Existing requirements can be found in HA regulations and guidelines [12, 13] for clinical drug development and computerised system validation; however additional controls should be considered to ensure data is traceable and integrity is maintained. Genomic data presents significant opportunity for reusability of the data for future insights, real world data studies and other analyses; therefore, it should be generated per FAIR data principles (Findable, Accessible, Interoperable and Reusable) where possible [14]. Contractual agreements between pharmaceutical sponsors and NGS service providers should establish FAIR data requirements.

Data controllers, processors and accountabilities should be traceable through a combination of contractual agreements, and data integrity controls (procedural and technical) throughout the data lifecycle (see also Sect. 4) [12, 15]. Documentation should objectively demonstrate the integrity of the data was maintained to support patient care and treatment, efficacy and/or other analysis decisions [9, 12, 13, 15].

## Clinical Quality Considerations: Data Quality

**Fig. 2:**
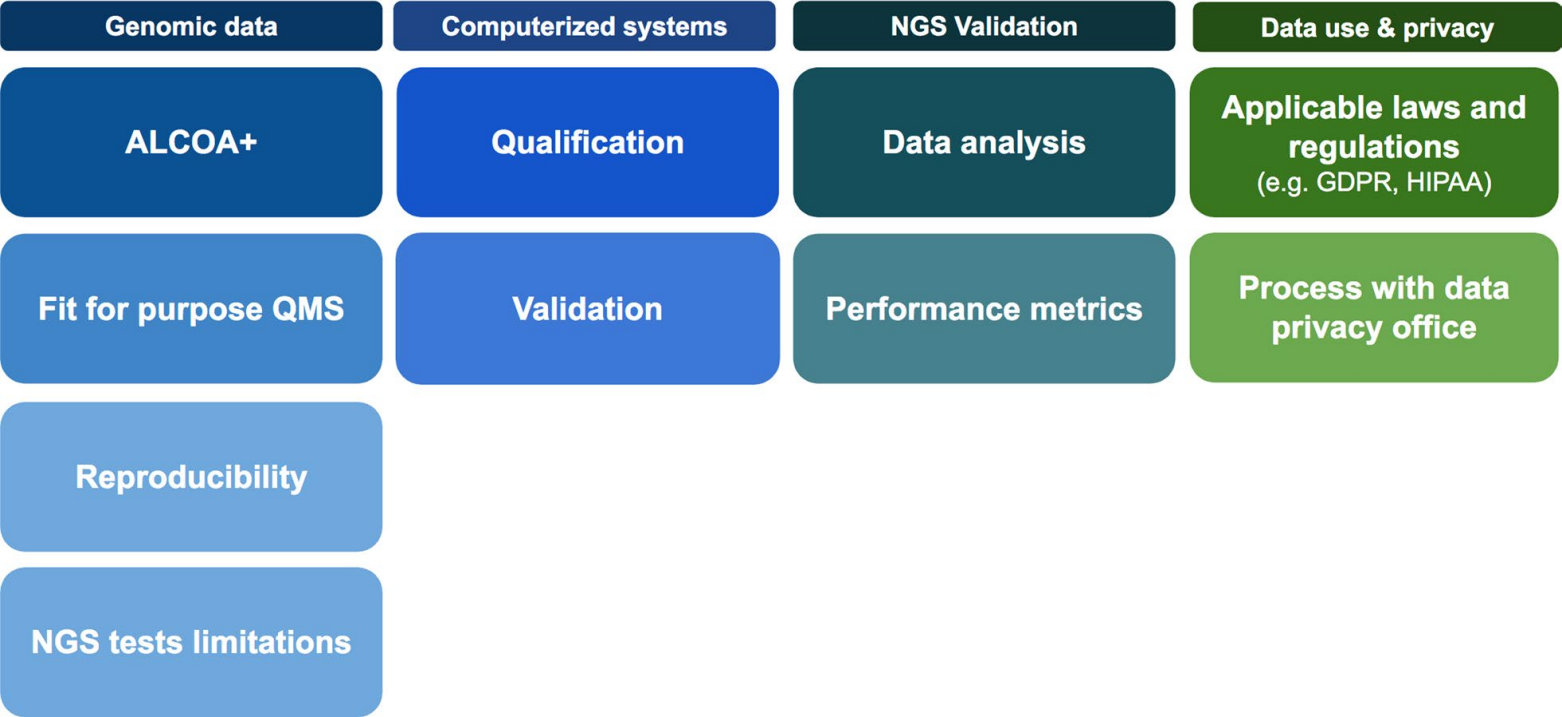
Data quality considerations

Data generated through NGS may support clinical decisions for patient care, patient eligibility for a clinical study, patients stratification and/or other research activities. This set of considerations is critical to ensure quality while using NGS service providers in clinical drug development. Requirements were already established in ICH and various HA regulations and ISO standards, but can be complemented by recommendations issued by medical genetics societies. The data clinical quality considerations are detailed below and summarized at the end of this section (Fig. 2).

### Genomic Data

To protect patient rights and wellbeing it is critical that the integrity of genomic data and derived insights provided to HCPs is maintained [9, 12, 16]. NGS-based tests are technically complex in that they have an end-to-end workflow consisting of multiple systems from sample processing and sequencing to a data analysis bioinformatics pipeline for reporting. This workflow includes multiple steps for ingestion of data, data generation, data transfer and ultimately data retention. Throughout this sample and data lifecycle ALCOA + principles (Accurate, Legible, Complete, Original, Attributable, Contemporaneous, Complete and Enduring) [17] should be considered to ensure the integrity of the data reported to the pharmaceutical sponsor, HCPs and HAs.

In summary, when genomic data are intended for submission to a HA or to be provided to a HCP, pharmaceutical sponsors should ensure genomic laboratories have:

- A defined and fit-for-purpose quality management system (QMS) and are accredited, if applicable, to ensure full NGS-based test workflow (from sample collection kit preparation to DNA extraction through to results interpretation) [9, 18–20]
- Well-defined and validated sample tracking processes to provide traceability of results to the patient [18–21]
- Processes in place to ensure scientifically justified analysis parameters are used and documented [18, 20]
- NGS Platforms, including both Laboratory and Computational Methods, that are validated for compliance against quality metrics [12, 15, 18–20]
- Computerised system validation and/or qualification policies, processes, controls and monitoring in place to ensure systems are fit for their intended use [9, 12, 15, 18]
- Audit trails implemented (as required) and assessed as fit for purpose and regularly reviewed [9, 12, 15]
- A data workflow, including data processing and analysis procedures clearly documented and followed. Raw sequencing requirements, post-sequencing bioinformatics pipeline updates and data management practices should be considered [9, 12, 15, 19]
- Adequate procedures or monitoring controls in place to demonstrate oversight and resolution of data integrity risks [9, 12, 15]
- Data archival procedures in place to ensure reproducibility of analyses [9, 12, 15]
- Use well defined or industry standard biomarker definitions, if available [19, 20, 22–24]
- Controls in place where test limitations exist (e.g., false positive/false negative rates of algorithms used), the impact on data is communicated to HCP and patients in a manner that supports clinical use of data [19, 25, 26]
- The ability to use widely accepted standard open file formats to support future use of the data [19, 20, 27]
- Controls in place to assess and address data standard requirements for data recipients. For example pharmaceutical sponsors may have specific requirements to facilitate useability. Additionally genomic data intended to be submitted to HA should comply with the applicable data standards, such as CDISC (Clinical Data Interchange Standards Consortium)

### Computerised Systems Used for NGS Activities

In clinical drug development, all GxP computerised systems (e.g., laboratory instruments, laboratory information management systems (LIMS), sample management systems, IT development tools) must be qualified or validated for their intended use [9, 12, 15]. This includes risk-based validation or qualification of the software, components and infrastructure that comprises the system or is used in the post sequencing analysis bioinformatics pipeline. Hence, these considerations would apply to NGS service providers like any other type of service provider used in a clinical trial.

### NGS Validation

Similarly, analytical test methods used in clinical drug development must also be validated for their intended use. Such analytical validation requires the careful prospective end-to-end study of appropriately justified method parameters and performance characteristics (e.g., accuracy, precision, limits of detection, specificity, robustness) from sampling through to data reporting. In order to demonstrate safety and effectiveness, developers of NGS-based tests must provide assurance of accurate and reliable detection of clinically relevant variants through careful design, development and analytical validation of such techniques [12, 13, 19, 20, 26].

NGS tests can be quite complex, involving many elements to achieve proper collection, and handling of clinical and laboratory samples, selection of adequate sampling materials, accurate laboratory analysis, and correct data processing through use of appropriate bioinformatics pipelines. Therefore, robust validation policies, processes, controls and monitoring must be in place to ensure NGS-based tests, including individual test elements and methods, that directly impact on the reliability of data are fit for purpose throughout the data processing and testing life cycle [9, 12, 13, 19, 20, 26].

In order to aid in the development of such robust controls, HA recommendations are available that outline key considerations for test design, development and validation [28]; Analytical standards for test development and performance characteristics for test validation (thresholds/definitions) must be well defined or based where possible on HA or medical Genetics Society standards [19] Additionally, processes must be in place to monitor ongoing performance characteristics (quality metrics) and ongoing validity of methods used for reproducible analytical results and classification of variants. [26, 28]

### Data Use & Privacy

Some NGS applications require the capture of a high volume of genomic data that is processed to generate valuable clinical insights [1–6]. Patient rights and privacy of personal data, including information collected must therefore be protected [19, 26, 29, 30]. All data collected and data created as a result of sample processing must be safeguarded, and its use must be in compliance with applicable national legislation such as Regulations on Management of Human Genetic Resources of the People's Republic of China, as well as regional data privacy legislation such as General Data Protection Regulation (GDPR) in the EU [29] and Health Insurance Portability and Accountability Act (HIPAA) in the United States [30].

While manufacturers are responsible for privacy controls for tests they develop and distribute, pharmaceutical sponsors must ensure NGS data use is disclosed and privacy risks are considered and addressed in clinical trial consents and commercial agreements [9, 26, 31]. When tests are co-developed data handling agreements need to be in place to address roles and responsibilities as it relates to applicable laws such as GDPR and HIPAA [29, 30]. Overall, pharmaceutical sponsors should establish a data privacy process to ensure that local privacy requirements (e.g., for recognizing and addressing data subject requests and withdrawal of consent, if applicable) are addressed. Finally, genomic sequencing should be performed within the scope of the protocol specifications, subject consent and contractual agreements [9, 26, 31, 32].

Manufacturers and pharmaceutical sponsors are responsible for ensuring genomic data is stored in a secure manner. Controls should be in place as required by applicable local laws to address security requirements, and access to data should be limited, not only to protect the integrity of the results, but also the privacy of the patients. These controls should be routinely reviewed against applicable local laws and security tests should be performed to assess weakness in security measures and to identify new threats [12, 13, 29, 30].

The return of genomic individual research results [33] and their potential reinterpretation [34] fall beyond the scope of clinical quality, hence were out of scope for this review. Processes that comply with applicable local and global laws and regulations are usually managed by data privacy and legal teams of pharmaceutical sponsors.

## Clinical Quality Consideration: Patients

Patient considerations are clearly stated in clinical trials regulations and HA applicable guidelines [9, 32, 35]; however, there were several guidances from medical genetics societies that provided additional considerations for patients undergoing genomic testing. We first reviewed the requirements for transparency to patients and their HCPs, and then the ones for patient consent.

### Transparency to Patients and Their HCPs

Identifying pathogenic variants through genomic testing can guide treatment recommendations [3, 4, 6]. Therefore, accurate, reliable genomic data and derived insights must be available to assist patients and their HCPs in making informed decisions on their next course of action (treatment, prophylactic measures, additional testing, etc.) [19, 26, 36].

Through the process of genomic testing, additional variants (e.g., germline pathogenic variants) or biomarkers (e.g., microsatellite instability (MSI) status) could be inferred using algorithmic methods [25]. Variants are also classified for their pathogenicity. These outcomes are frequently reported to HCPs and may be used to inform decisions on the next course of action [25, 26]. To protect patient rights, well-being and safety, methods to generate molecular sequencing data and their directly derived variables, should be generated, stored, analyzed and reported in a consistent, transparent and controlled manner [26, 36, 37]. To ensure transparency of communication with HCPs and patients, the above considerations can be translated into the following clinical quality recommendations:

- Processes, controls and monitoring are in place to ensure that generation of sequencing data and derived variables are validated [9, 25, 26];
- The pharmaceutical sponsors have oversight on methods being verified (and reviewed on an ongoing basis) for analytical performance metrics (e.g., accuracy, precision, reproducibility) and compliance [37];
- Contextual information, test limitations and potential risks (e.g., risk of false positive, incidental findings) are communicated in a timely and transparent manner to the patient and their HCPs [26, 38].

### Patients Consent

Patients participating in clinical trials must be provided with written information and consent forms that have been approved by an IRB/IEC [9]. For consent to be valid, it must be voluntary and informed, and the person consenting must have the capacity to make the decision—these principles are clearly defined in ICH-GCP and well established in the context of clinical trials [9, 31, 32]. However, there might be local requirements for consent in commercial and/or research settings that must be also met [19, 26, 39]. Both regulatory agencies and genetic medical societies have highlighted that HCPs and clinical investigators should be provided with information to ensure that patients understand the potential benefits and risks to their health associated with genomic testing and the planned data uses (primary and secondary) [19, 26, 39]. Patient’s rights (including privacy) with regards to their medical (genomics) data must be safeguarded [19, 26, 39].

For genomic testing, whether used in clinical trials or in commercial settings, the above considerations can be transcribed into the following recommendations—to ensure patient’s rights, well-being and safety are protected:

- Consent form development ensures inclusion of sufficient NGS testing and data collection disclosure information, including the breadth of testing, use of data (including additional data collected or secondary uses), potential use of data by third parties, and any unused and/or unreported sequencing data, that participation is voluntary, and that the subject may choose not to participate or may withdraw at any time [9, 19, 29, 31, 38, 39]
- Implications (e.g., prophylactic measures) of discovering a potential pathogenic germline variant are explained and contextualized [19, 25, 39]
- Storing anonymized patients data and secondary use of data (e.g., research) is clearly stated and explained [31, 40]
- Rights for patient privacy, withdrawal of consent (e.g., for secondary use of data) are clearly stated and explained [9, 39]
- Disclosure of limitations of data (and clinical significance of limitations) and explanations provided as applicable [26]

## Clinical Quality Consideration: Sponsors Oversight

Requirements for oversight of NGS service providers do not differ from the ones applicable to other types of service providers (e.g., central laboratories, independent reading facilities, electronic trial system vendors) used in clinical drug development. Pharmaceutical sponsors retain the ultimate responsibility for the GxP activities conducted, as well as the quality and integrity of trial data generated on their behalf [9]. Hence, all considerations reviewed in this section were already well established and described in HA regulations [32], in ICH [9] and ISO standards [18].

In clinical trials, NGS service providers can perform various levels of GxP type activities under different types of relationships such as a standard service provider relationship, co-development relationship or collaborative model relationship. Irrespective of the relationship type the pharmaceutical sponsors must ensure clear oversight of the GxP service provider (and their third parties) including assurance they are fulfilling contractual obligations and regulatory requirements to ensure end-to-end sequencing data integrity and data credibility [9, 15, 18].

NGS service providers used by pharmaceutical sponsors must be qualified to perform contracted activities. The selection process shall be documented to demonstrate credentials of the provider. Contracts and agreements should reflect the expected quality of the services based on applicable local and national laws and regulations, including national or regional medical laboratory accreditation, where required [41]. Such contracts and agreements should also include disclosures of intent to subcontract and expectations for oversight of third parties. Agreements should address data processing and the pharmaceutical sponsors requirements for data handling and data quality at the Service Provider. Once contracts are in place, pharmaceutical sponsors shall have processes and procedures in place to ensure the service provider is overseen throughout the performance of contracted activities and issues are addressed in a timely manner. Requirements and oversight activities should be documented and available for HA inspection. A risk-based approach should be taken for supplier/service provider audits. Audits are to be performed in and documented in accordance to ICH-GCP [9] and other applicable regulations. Of note, there is also a recent effort to shift clinical quality assurance practices across the GxP areas [42–47]: these approaches which leverage data analytics would be well suited to identifying and assessing clinical quality risks for NGS.

## Conclusion

In this paper, we reviewed applicable HA requirements, international standards and recommendations from medical genetics societies to identify relevant clinical quality considerations to ensure data integrity and patient safety in clinical drug development using NGS service providers.

When using NGS in clinical trials it is recommended the quality considerations in this review be evaluated and applied in a fit for purpose manner by pharmaceutical sponsor clinical quality organizations to strengthen the ability to:

- Demonstrate that data integrity was maintained through well defined, documented and understood quality standards for genomic tests
- Provide assurance to patients that their data is safeguarded and is used in an ethical and responsible way
- Demonstrate that developed tests lead to better patient outcomes and increased patient safety through generation of transparent, accurate and reliable genomic results to assist HCPs in making informed decisions on next course of action
- Demonstrate patient rights were maintained, and sufficient controls were in place to identify and address quality implications, which could have an effect on patient care (diagnosis, treatment, etc.)
- Demonstrate that up-to-date information on the technology was disclosed to healthcare professionals, including clear communication of limitations of the data (e.g., false positive / false negative rates) so they can confidently use the data to guide clinical decisions

Clinical quality assurance for NGS service providers will continue to be essential to ensure data integrity and patient safety as NGS testing is well established to guide clinical trials [6] while liquid biopsy is being used increasingly for clinical trial recruitment [48]. This work could be used as a basis to develop a set of guidelines for ensuring NGS clinical quality.

## Supplementary Information

Below is the link to the electronic supplementary material.

Supplementary file1 (PDF 115 kb)
