# Supplementary material for: Clinical Quality Considerations when Using Next-Generation Sequencing (NGS) in Clinical Drug Development
Source: Ther Innov Regul Sci. 2021 May 27;55(5):1066–74. doi: 10.1007/s43441-021-00308-6 (PMC8332578; doi:10.1007/s43441-021-00308-6)
Supplement: Supplementary file 1 — Supplementary file1 (PDF 115 kb) [file 43441_2021_308_MOESM1_ESM.pdf]

Clinical quality considerations when using Next-Generation Sequencing (NGS) in clinical drug development

Supplementary Material

Timothé Ménard, PharmD[1] **• Alaina Barros**[2] **• Christopher Ganter**[2]

1. F. Hoffmann-La Roche AG, Basel, Switzerland
   Correspondence to timothe.menard@roche.com
2. Genentech Inc. - A Member of the Roche Group, South San Francisco, USA

Search strategy

While this paper was not intended to be a systematic review, we applied the following strategy and criteria to screen and identify relevant regulations, standards and scientific journals.

- We focused on standards and regulations applicable to the International Conference on Harmonization (ICH) regions (i.e., United States of America (USA), European Union (EU) and Japan).
- From the ICH guidelines, we selected the one applicable to clinical drug development (i.e., which was the scope of this review) - ICH-E6 Good Clinical Practices
- From ISO standards, we considered the ones relevant for Quality Management Systems (QMS) and the use of medical devices.
- We reviewed major HA regulations and guidance, and selected the ones that pertain to the development, use and validation of NGS in the context of use in clinical trials.
- We also included major HA regulations applicable to clinical drug development, for example the US Code of Federal Regulations (CFR) that encompass requirements for quality management, use of data and validation of electronic systems in clinical trials.
- Finally, we reviewed a selection of genetics scientific journals for relevant articles and reviews that could inform clinical quality considerations. We only considered scientific journals where institutions (such as the American College of Medical Genetics) or scientific working groups were known to have discussed or issued recommendations regarding use of NGS in clinical practice.
- As explained in the Background section of the manuscript, we limited our search up to 2010 as NGS was an emerging technology and did not want to include outdated information.
- A list of standards, regulations and journals included for review can be found in the table below.

List of standards, regulations and scientific journals

Materials	Search criteria	Links
International Conference of Harmonization (ICH) Guidelines	Applicability to clinical drug development (i.e., ICH-E2(R2))	https://www.ich.org/
ISO (International Organization for Standardization) standards	Applicability to quality management in clinical drug development and/or medical devices (i.e., ISO 13485)	https://www.iso.org/home.html
Health Authorities regulations and guidance on clinical drug development and/or Next-Generation Sequencing	Applicability to clinical drug development and encompasses quality management, use of data and validation of electronic systems and/or NGS<br><br>ICH regions (i.e., USA, EU, Japan)	https://www.ema.europa.eu/en/human-regulatory/research-development/scientific-guidelines<br><br>https://ec.europa.eu/health/<br><br>https://gdpr-info.eu/<br><br>https://www.ecfr.gov/cgi-bin/ECFR?page=browse<br><br>https://www.fda.gov/regulatory-information/search-fda-guidance-documents<br><br>https://www.hhs.gov/hipaa/index.html<br><br>http://www.jpma.or.jp/english/parj/pdf/2020.pdf
Scientific peer-reviewed journals	Should be referenced as journals of choice where institutions (e.g. the American College of Medical Genetics, European Society of Human Genetics) or scientific working groups discussed or issued recommendations regarding use of NGS in clinical practice	https://www.acmg.net/ACMG/Medical-Genetics-Practice-Resources/Genetics_In_Medicine.aspx<br><br>http://varnomen.hgvs.org/recommendations/general/<br><br>https://www.nature.com/ejhg/about<br><br>https://www.jmdjournal.org/<br><br>https://www.jpatholtm.org/about/index.php
